# A comparative analysis of three graph neural network models for predicting axillary lymph node metastasis in early-stage breast cancer

**DOI:** 10.1038/s41598-025-97257-z

**Published:** 2025-04-22

**Authors:** Enock Adjei Agyekum, Wentao Kong, Yong-zhen Ren, Eliasu Issaka, Josephine Baffoe, Wang Xian, Gongxun Tan, Chunjing Xiong, Zhangye Wang, Xiaoqin Qian, Xiangjun Shen

**Affiliations:** 1 Department of Ultrasound Medicine, Affiliated People’s Hospital of Jiangsu University, Zhenjiang, Jiangsu China; 2 School of Computer Science and Communication Engineering, Jiangsu University, Zhenjiang, Jiangsu China; 3 Department of Ultrasound Medicine, Nanjing Drum Tower Hospital, Affiliated Hospital of Medical School, Nanjing University, Nanjing, China; 4 Northern Jiangsu People’s Hospital, Northern Jiangsu People’s Hospital Affiliated to Yangzhou University, The Yangzhou Clinical Medical College of Xuzhou Medical University, The Yangzhou Clinical Medical College of Jiangsu University, Yangzhou, Jiangsu China; 5 College of Engineering, Birmingham City University, Birmingham, B4 7XG UK; 6 School of Automotive and Traffic Engineering, Jiangsu University, Zhenjiang, 212013 P.R. China

**Keywords:** Axillary lymph node metastasis, Breast cancer, Graph neural network, Graph convolutional network, Graph attention network, Graph isomorphism network., Medical research, Breast cancer, Oncology, Cancer, Diseases, Cancer

## Abstract

The presence of axillary lymph node metastasis (ALNM) in breast cancer patients is an important factor in deciding whether to have axillary surgery or pursue alternative treatments. Based on axillary ultrasound (US) and histopathologic data, three graph neural network models were compared to predict ALNM in early-stage breast cancer. The patients were randomly divided into two data sets: training (80%) and testing (20%). Predictive performance was measured using accuracy, sensitivity, specificity, positive predictive value, negative predictive value, F1 score, and area under the curve (AUC). In the test cohort, the graph convolutional network (GCN) performed the best in predicting ALNM, with an AUC of 0.77 (95% confidence interval [CI]: 0.69–0.84). In conclusion, the GCN model has the potential to provide a noninvasive tool for detecting ALNM and can aid in clinical decision-making. Prospective studies are expected to provide high-level evidence for clinical usage in future investigations.

**Supplementary Information:**

The online version contains supplementary material available at 10.1038/s41598-025-97257-z.

## Introduction

As per the Global Cancer Statistics 2020, female breast cancer is the most common malignant tumour with the fifth highest global fatality rate among all cancer types^1^. Accurately determining the status of axillary lymph nodes (ALNs) is essential for clinical staging, optimizing treatment, and assessing prognosis^2,3^.

With almost 70% of lymphatic drainage from the breast moving through ALNs, they are the main sites of lymphatic metastases for patients with breast cancer.

The gold standard for determining axillary lymph node metastasis (ALNM) is axillary lymph node dissection (ALND). On the other hand, ALND is an invasive process that may result in surgical complications^4,5^. The current gold standard for ALN staging is sentinel lymph node biopsy (SLNB), which directs the surgeon’s decision for the course of treatment and helps the physician decide whether to undertake ALND^6,7^. However, because SLNB and ALND are both invasive procedures, there is a chance that they will have undesirable side effects, such as upper limb edema and arm numbness, which would significantly lower the patient’s quality of life^8–11^.

Besides the diagnostic performance of current non-invasive imaging modalities to assess ALNM, including axillary ultrasound (US), is limited by their high false-negative rates^12^.

Artificial intelligence (AI) is rapidly gaining traction in medical imaging and in this AI-driven era, radiology advancements are centred on improving decision support systems to maximize the benefits of non-invasive imaging procedures. Radiology’s vast digital data sets make it ideal for AI^13^. Machine learning, a substantial subset of AI, plays an important supportive role in enhancing diagnostic and prognostic accuracy^14^.

Deep learning^15^, a branch of machine learning, is a unique approach that uses end-to-end learning to autonomously reveal many layers of representation tailored to certain prediction tasks. Designed to infer graph-described data, graph neural networks (GNNs) are a kind of deep learning algorithm^16^ that can provide a simple technique to solve prediction tasks at the node, edge, and graph levels^17^. Graph-based models have proven promising outcomes in several computer vision applications that combine nodes and their connections^18^.

The study’s first goal is to create GNN-based models utilizing three different GNNs to classify ALNM from non-ALNM based on axillary US and clinicopathologic data. The second goal is to evaluate the diagnostic performance of the models. Third, compare the performances of the models.

## Materials and methods

### Patients

This study utilized patient data from a previously published study by Zheng et al.^19^, which included clinical, histopathologic, and axillary US findings for each patient. The original study was conducted in accordance with the principles of the Declaration of Helsinki and received ethical approval from the Institutional Review Board of Sun Yat-sen University Cancer Center and written informed consent was obtained from all participants. The dataset was made available under the Creative Commons Attribution 4.0 International (CC BY 4.0) license (https://creativecommons.org/licenses/by/4.0/), which permits unrestricted use, distribution, and reproduction, provided appropriate credit is given.

The data were used in compliance with the specified terms, ensuring full anonymization and strict confidentiality in handling. No additional ethical approval was required for this secondary analysis. The dataset comprised 1,342 women with 1,342 breast lesions examined between January 2016 and April 2019. Of these, 584 women (mean age: 50 years; range: 26–83 years) with 584 malignant breast lesions were included in the final analysis.

**Fig. 1:**
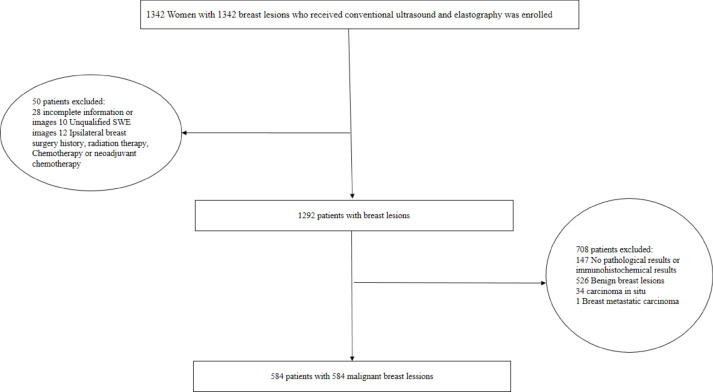
Schematic diagram of patient selection.

The flowchart shown in Fig. 1 describes the patient recruitment process. SLND or ALN dissection results showed that 247 had ALNM and 337 had non-ALNM. Table 1 shows the inclusion and exclusion criteria.

**Table 1:** Inclusion and exclusion criteria.

Inclusion and exclusion criteria
Inclusion criteria
1. Women with ultrasound-suspected breast masses
2. Availability of clinical data
3. Patients who underwent breast surgery and sentinel lymph-node biopsy or axillary lymph node dissection with curative intent
Exclusion criteria
1. Preoperative therapy (resection biopsy, neoadjuvant radiotherapy or chemotherapy)
2. Patients with multifocal lesions or bilateral disease
3. Masses deeper than 3 cm in depth due to the attenuation of SWE or larger than 3.5 cm in diameter due to the limited width of the ultrasound probe
4. Unqualified 2D- shear wave elastography measurements, which means little or no shear wave signal was acquired in the ROI of shear wave elastography
5. Benign breast lesions or carcinoma in situ
6. Missing important histopathological results (immunohistochemical results or lymph-node results)
7. Incomplete information.

Histopathologic results of the breast cancer included tumour type, estrogen receptor (ER) status, progesterone receptor (PR) status, human epidermal growth factor receptor-2 (HER-2), and Ki-67 proliferation index. Clinical data included patients’ age, US size, tumour location, and BI-RADS category. Axillary US findings included the ratio of long-axis diameter to short-axis diameter < 2, diffuse cortical thickening > 3 mm, focal cortical bulge > 3 mm, eccentric cortical thickening > 3 mm, complete or partial effacement of the fatty hilum, rounded hypoechoic node, complete or partial effacement of the fatty hilum, nonhilar cortical blood flow on colour Doppler images, complete or partial replacement of the node with an ill-defined or irregular mass and microcalcifications in the node.

### Data preprocessing and construction of the graph

The enrolled patients were randomly separated into two groups: the training cohort and the independent test cohort, with a 4:1 ratio. Univariate logistic regression analysis was utilized in the training cohort to identify candidate factors based on clinical, histopathologic, and axillary US findings. A standard scalar was used to standardize the candidate factors.

To create the graph, a feature table with 466 rows and 12 columns for the training cohort and 118 rows and 12 columns for the independent test cohort was created first. The 12 columns had ten candidate factors, one unique ID for each row, and the target class. Each row was handled as a single node (*v*). An edge table (*E*) was created by computing the cosine similarity between rows. Notably, the relational edge table includes several connections (edges) between nodes. The presence of so many edges may contribute to noise, redundancy, sometimes unnecessary information, and increased complexity in the model^20^.

To decrease the number of edges and increase the robustness of the model, we took into consideration in this study a correlation cutoff value of ≥ 0.95 for node connections. While enhancing performance, raising the threshold values decreases the number of edges. This quantifies the relationships between the patients based on the distinctive characteristics of each patient. Examples of how the link is established by calculating the similarity scores of each row include nodes 1 to 2, nodes 1 to 3, nodes 1 to 466, nodes 2 to 3, nodes 2 to 4, and nodes 2 to 466. Each time, the cosine similarity of two nodes is measured, and a relationship between a single node and all other nodes is identified.

With the set of nodes (*v*_*n*_) and edges (*e*_*m*_), a graph *G* = (*V*,* E*) is generated where *v*_*n*_ ∈ *V* and *e*_*n*_ ∈ *E*, *n*, *m* denote the number of nodes and edges, respectively. In the graph, the edges are constructed as *eij* = (*vi*,* vj*), which represents the relationship between nodes *vi* and *vj*. An adjacency matrix (A) is also generated from the graph (G) with n × n dimensions. If *Aij* = 1, there is an edge between two nodes (*eij* ∈ *E*), and *Aij* = 0 if *eij* ∉ E.

**Fig. 2:**
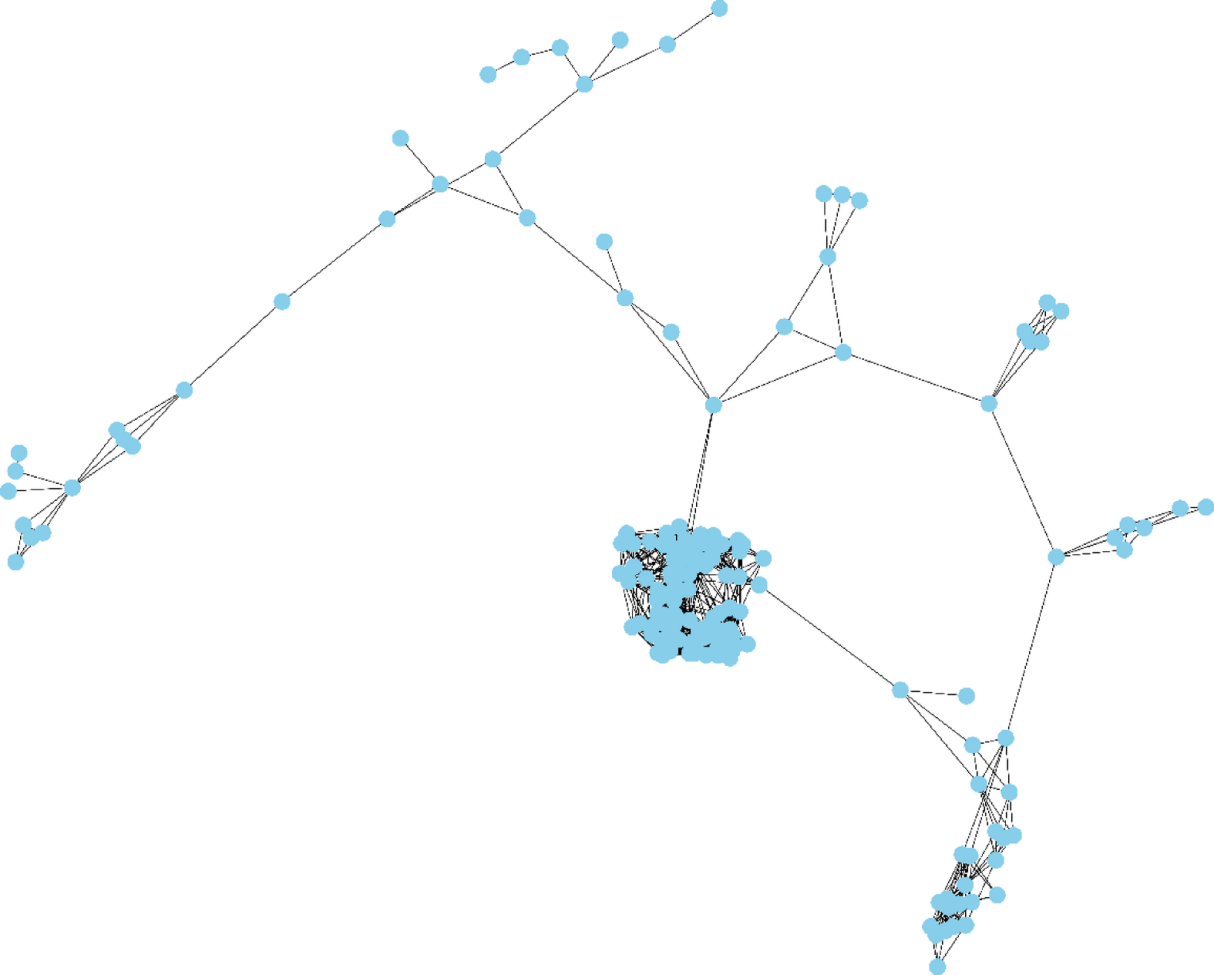
A visualization of the graph in the training cohort.

**Fig. 3:**
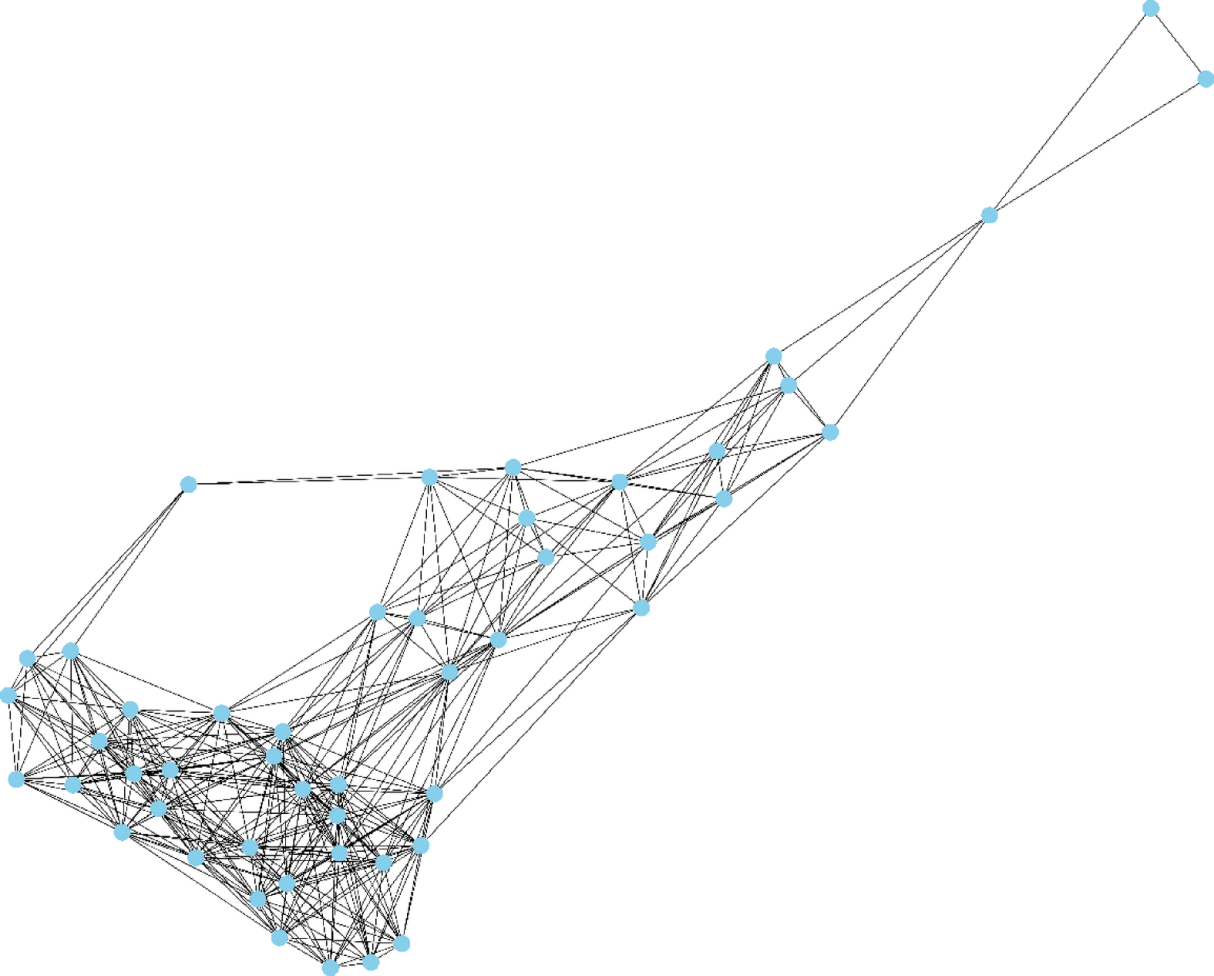
A visualization of the graph in the test cohort.

The 10 candidate factors, excluding the unique target classes, make up the graph’s feature vector X, where *Xv* stands for the feature vector of the particular node (*v*)^21^. The relationships between patients serve as the foundation for the created graph. The resultant graph is complex and large. A small portion of the graph is depicted in the Figs. 2 and 3.

### Construction of the graph neural network models

We created our models using the GNN, which included a graph convolutional network (GCN), a graph attention network (GAT), and a graph isomorphism network (GIN).

GCN extends convolutions from the Euclidean domain to the graph domain, which is defined by data structured as nodes and edges and was first described by Kipf and Welling^22^. It propagates information throughout the graph and aggregates it to update node representations^23^. The spatial technique uses a setup in which operation objects are non-fixed in size, similar to how convolutional layers in convolutional neural networks (CNNs) aggregate local information in images. Each GCN layer computes new node representations depending on its current characteristics and those of its neighbours^24,25^.

Veličković et al.^26^, proposed GAT, which extends GCNs by incorporating an attention mechanism to weigh the value of nearby nodes. This method is based on the transformer model’s attention, which allows the network to focus on several areas of the input at the same time. The two main steps in the local function that generates the GAT update rule are (1) computing attention scores for each pair of nodes in the graph, which show how relevant neighbour nodes’ features are to a particular central node; and (2) aggregating the features of the central node and its neighbours into a weighted sum, where the weights are determined by the attention scores that were previously computed^26^.

GIN uses a sum aggregation function and a multi-layer perceptron (MLP) to analyze node characteristics, which increases their expressiveness when compared to earlier models^27^. GINs are built on the premise that they should be able to discriminate between non-isomorphic networks, a quality that prior GNN models fell short of since their message-passing methods are inadequately discriminative^26,28^.

GINs convert features using a sum aggregation function and a trained MLP. GINs’ construction is influenced by the Weisfeiler-Lehman graph isomorphism test, which ensures they can theoretically discriminate between any two non-isomorphic graphs^29^. As a result, GINs provide improved performance in tasks like graph and node classification^27^.

One-hot encoding was used as the label to reflect the ALN status of the breast cancer patients. The created graphs were input into the network to update the model parameters throughout the training phase. The network outputs are classification results, and the cross-entropy between the outputs and labels is used to calculate the loss function.

To update the model parameters, the Adam optimizer was used with a batch size of 32 and a learning rate of 0.0001 based on previous research and their balance of computing efficiency and training stability was chosen. A learning rate of 0.0001 ensures smooth convergence, while the batch size of 32 allows for steady gradient updates without consuming too much memory. PyTorch 2.2.2 was used to implement the training and testing codes and Keras version 2.10.0 utilizing Python (version 3.10.12). The models underwent 1000 epochs of training to prevent overfitting.

### Evaluation metrics employed in this study

A detailed description is provided in the supplementary method. Metrics including the area under the curve (AUC), sensitivity (SEN), specificity (SPEC), accuracy (ACC), F1 score, negative predictive value (NPV), positive predictive value (PPV), and other widely used clinical statistics were employed to evaluate the models’ performance on the training and testing datasets.

### Statistical analysis

IBM SPSS Statistics for Windows version 26.0 (Armonk, New York, USA) and Python 3.10.12 were used for the statistical analysis. Pearson’s chi-square test or Fisher’s exact test was used to compare categorical characteristics. The independent sample t-test was used for continuous variables with a normal distribution, whereas the Mann-Whitney U test was used for those without. A two-sided P value of < 0.05 indicated a statistically significant difference.

## Results

### Clinical characteristics

Table 2 shows the clinical characteristics of 584 breast cancer patients from both the training and independent test cohorts. The training cohort and test cohort had ALN metastatic rates of 42.3% and 42.4%, respectively. The training cohort had 466 patients (mean age 50.46 years ± 10.36), while the test cohort had 118 patients (mean age 49.52 years ± 10.20).

**Table 2:** Participant and tumor characteristics.

Characteristics	Training (466)	Test(118)
Age, mean ± SD, years	50.46 ± 10.36	49.52 ± 10.20
US size, mean ± SD, mm	19.06 ± 6.57	18.13 ± 6.10
HER2		
Positive	104 (22.3%)	31 (26.3%)
Negative	362 (77.7%)	87 (73.7%)
Ki-67		
Positive	396 (85.0%)	95 (80.5%)
Negative	70 (15.0%)	23 (19.5%)
PR		
Positive	338 (72.5%)	91 (77.1%)
Negative	128 (27.5%)	27 (22.9%)
ER		
Positive	372 (79.8%)	99 (83.9%)
Negative	94 (20.2%)	19 (16.1%)
Location		
Upper inner quadrant	126 (27%)	26 (22.9%)
Lower inner quadrant	65 (13.9%)	12 (10.2%)
Lower lateral quadrant	80 (17.2%)	24 (20.3%)
Upper lateral quadrant	195 (41.8%)	56 (47.5%)
Tumor type		
Invasive ductal carcinoma	412 (88.4%)	104 (88.1%)
Invasive lobular carcinoma	12 (2.6%)	6 (5.1%)
Other tumor types	42 (9.0%)	8 (6.8%)
BI-RADS category		
4A category	24 (5.1%)	7 (5.9%)
4B category	128 (27.5%)	36 (30.5%)
4C category	210 (45.1%)	57 (48.3%)
5 category	104 (22.3%)	18 (15.3%)
Different axillary US findings		
Term1		
Positive	177 (38.0%)	42 (35.6)
Negative	289 (62.0%)	76 (64.4%)
Term2		
Positive	31 (6.7%)	7 (5.9%)
Negative	435 (93.3%)	111 (94.1%)
Term3		
Positive	77 (16.5%)	17 (14.4%)
Negative	389 (83.5%)	101 (85.6%)
Term4		
Positive	127 (27.3%)	36 (30.5%)
Negative	339 (72.7%)	82 (69.5%)
Term5		
Positive	91 (19.5%)	20 (16.9%)
Negative	375 (80.5%)	98 (83.1%)
Term6		
Positive	35 (7.5%)	10 (8.5%)
Negative	431 (92.5%)	108 (91.5%)
Term7		
Positive	13 (2.8%)	5 (4.2%)
Negative	453 (97.2%)	113 (95.8%)
Term8		
Positive	0 (0%)	2 (2%)
Negative	466 (100%)	116 (98.2%)
Term9		
Positive	81 (17.4%)	17 (14.4%)
Negative	385 (82.6%)	101 (85.6%)
ALN metastasis		
Positive	197 (42.3)	50 (42.4%)
Negative	269 (57.7%)	68 (57.6%)

BI-RADS, Breast Imaging-Reporting and Data System; PR, progesterone receptor; ER, estrogen receptor, HER2, human epidermal growth factor receptor-2; US, Ultrasound; ALN, *axillary lymph node*;Term1, ratio of long axis diameter to short axis diameter<2; Term2, diffuse cortical thickening>3 mm; Term3, focal cortical bulge >3 mm; Term4, eccentric cortical thickening >3 mm; Term5, complete or partial effacement of the fatty hilum; Term6, rounded hypoechoic node; Term7, complete or partial replacement of the node with an ill-defined or irregular mass; Term8, nonhilar cortical blood flow on color Doppler images.

### Diagnostic performance of the three-graph neural network algorithms

A total of 18 parameters were gathered from each patient. After applying univariate logistic regression (Table 3), the parameters were reduced to ten, which were then used to create the graphs that serve as model inputs. The diagnostic performance of the three GNN models was based on size, location, and axillary US findings including the ratio of long axis diameter to short axis diameter < 2; diffuse cortical thickening > 3 mm; focal cortical bulge > 3 mm; eccentric cortical thickening > 3 mm; complete or partial effacement of the fatty hilum; rounded hypoechoic node; complete or partial replacement of the node with an ill-defined or irregular mass; nonhilar cortical blood flow on colour Doppler images.

**Table 3:** Univariate logistic regression analysis of ALN status in the training cohort.

Characteristic	Coefficient	Odds ratio (95% CI)	*P* value
Age	0.0010	1.0010 (0.9834–1.0190)	0.905
Location	0.1788	1.1958 (1.0313–1.3893)	0.019*
Size	0.0575	1.0591 (1.0284–1.092)	< 0.001*
Tumor type	0.0152	1.015 (0.8693–1.1814 )	0.845
BIRAD category	− 0.1867	0.8297 (0.7095–0.9692)	0.019*
ER	0.0003	1.00003 (0.9957–1.0049)	0.902
PR	− 0.0024	0.9976 (0.9930–1.0023)	0.319
Ki67	0.0033	1.0033 (0.9947–1.0121)	0.449
HER2	0.1766	1.1932 (0.9580–1.4859)	0.114
Term1	0.9057	2.4737 (1.6885–3.6410)	< 0.001*
Term2	2.6872	14.6903 (5.1043–62.0983)	< 0.001*
Term3	1.1147	3.0486 (1.8431–5.1395)	< 0.001*
Term4	0.6735	1.9611 (1.2999–2.9694)	< 0.001*
Term5	2.4585	11.6875( 6.5430–22.3184)	< 0.001*
Term6	2.0237	7.5664 (3.2880–20.5372)	< 0.001*
Term7	2.0663	7.8952 (2.0891–51.4087)	0.008*
Term8	NA	0.732342 (NA)	0.094
Term9	1.4958	4.4629 (2.6712–7.6777)	<0.001*

BI-RADS, Breast Imaging-Reporting and Data System; PR, progesterone receptor; ER, estrogen receptor, HER2, human epidermal growth factor receptor-2;Term1, ratio of long axis diameter to short axis diameter<2; Term2, diffuse cortical thickening>3 mm; Term3, focal cortical bulge >3 mm; Term4, eccentric cortical thickening >3 mm; Term5, complete or partial effacement of the fatty hilum; Term6, rounded hypoechoic node; Term7, complete or partial replacement of the node with an ill-defined or irregular mass; Term8, microcalcification in the node; Term9, nonhilar cortical blood flow on color Doppler images; NA, not applicable.

**Fig. 4:**
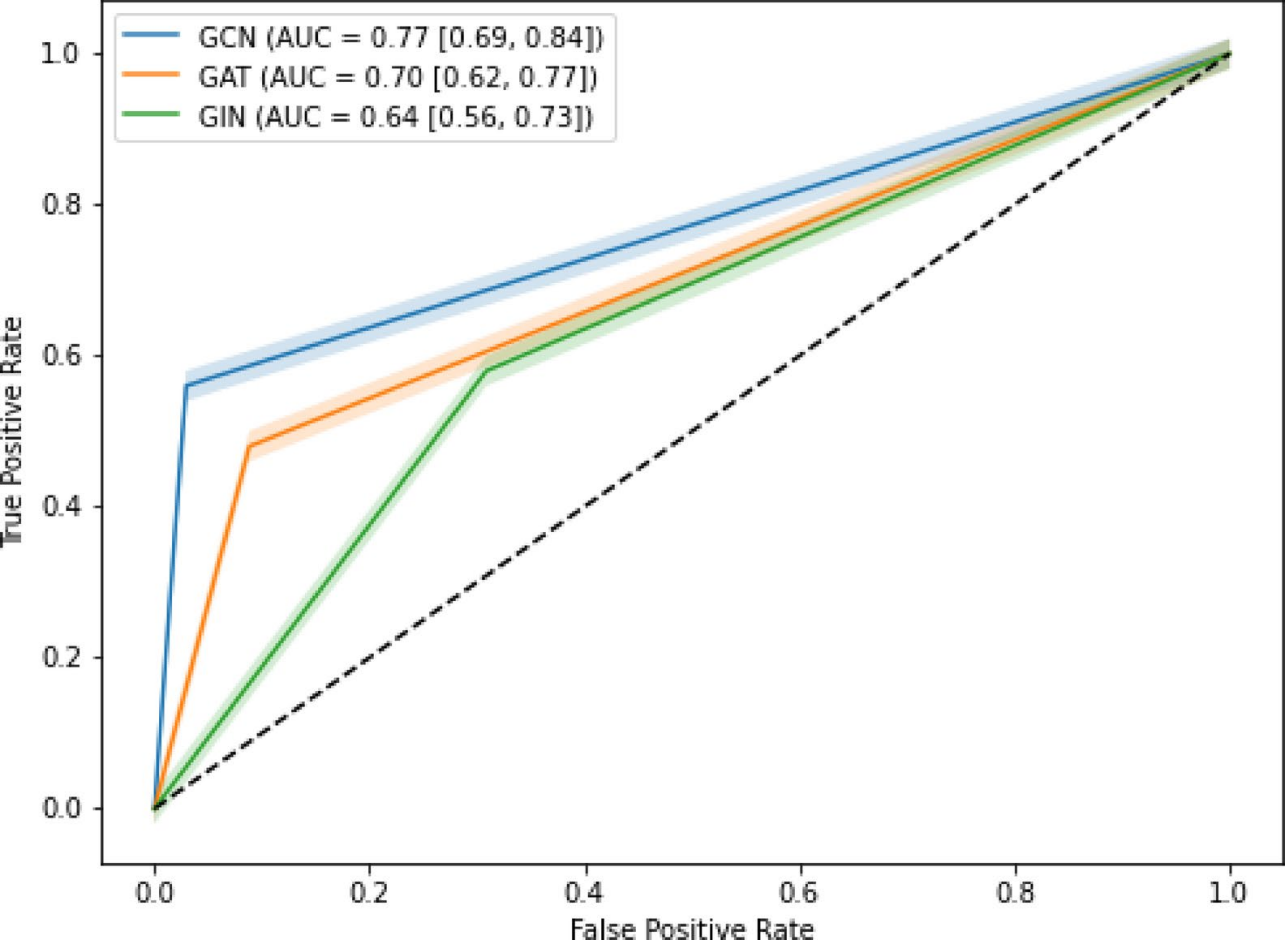
ROC curves of the models. ROC, receiver operating characteristic. (**A**) GCN (**B**) GAT (**C**) GIN.

In the test cohort, AUCs for GCN, GAT, and GIN were 0.77 (95% confidence interval [CI]: 0.69–0.84), 0.70(0.62–0.77), and 0.64(0.54–0.72), respectively (Table 4). The receiver operating characteristic (ROC) curves for each model are shown in Fig. 4. Thus, the GCN algorithm outperformed other GNN algorithms in the test cohort. When the three GNN algorithms were applied in the test cohorts, the GCN algorithm had a higher ACC (0.80) than the GAT (ACC: 0.73) and the GIN (ACC: 0.64). The GCN model’s SEN, SPEC, PPV, NPV, and F1 values in the test cohort were 0.56, 0.97, 0.93, 0.75, and 0.70, respectively (Table 4). The GAT model in the test cohort had SEN, SPEC, PPV, NPV, and F1 values of 0.48, 0.91, 0.80, 0.70, and 0.60, respectively (Table 4). In the test cohort, the GIN model had a SEN of 0.58, SPEC of 0.69, PPV of 0.58, NPV of 0.69, and F1 of 0.58 (Table 4).

**Table 4:** Performance summary of different models for prediction of ALNM.

	GCN	GAT	GIN
ACC	0.80 [0.73–0.87]	0.73 [0.65–0.81]	0.64 [0.56–0.73]
AUC	0.77 [ 0.69–0.84]	0.70 [0.62–0.77]	0.64 [0.54–0.72]
SEN	0.56 [0.47–0.65]	0.48 [0.39–0.57]	0.58 [0.49–0.67]
SPEC	0.97 [0.94–1.00]	0.91 [0.86–0.96]	0.69 [0.61–0.77]
PPV	0.93 [0.89–0.98]	0.80 [0.73–0.87]	0.58 [0.49–0.67]
NPV	0.75 [0.67–0.82]	0.70 [0.62–0.79]	0.69 [0.61–0.77]
F1	0.70 [0.62–0.78]	0.60 [0.51–0.69]	0.58 [0.49–0.67]

GCN, graph convolutional network; GAT, graph attention network; GIN, graph isomorphism network; AUC, area under the curve; ACC, accuracy; SEN, sensitivity; SPEC, specificity; NPV, negative predictive value; PPV, positive predictive value.

**Fig. 5:**
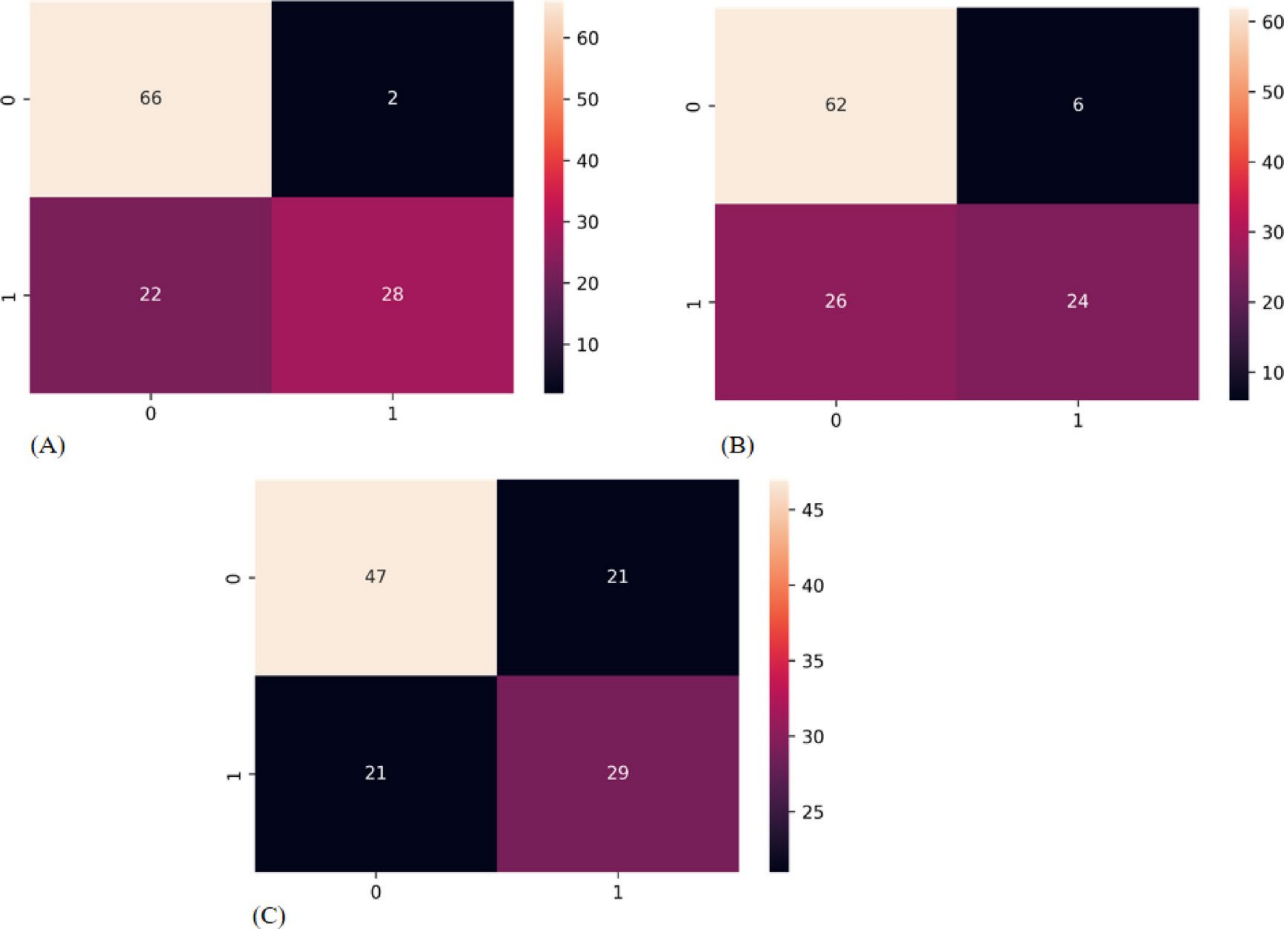
Confusion matrix. The 2 × 2 contingency table reports the number of true positives, false positives, false negatives, and true negatives: (**A**) GCN (**B**) GAT (**C**) GIN.

**Fig. 6:**
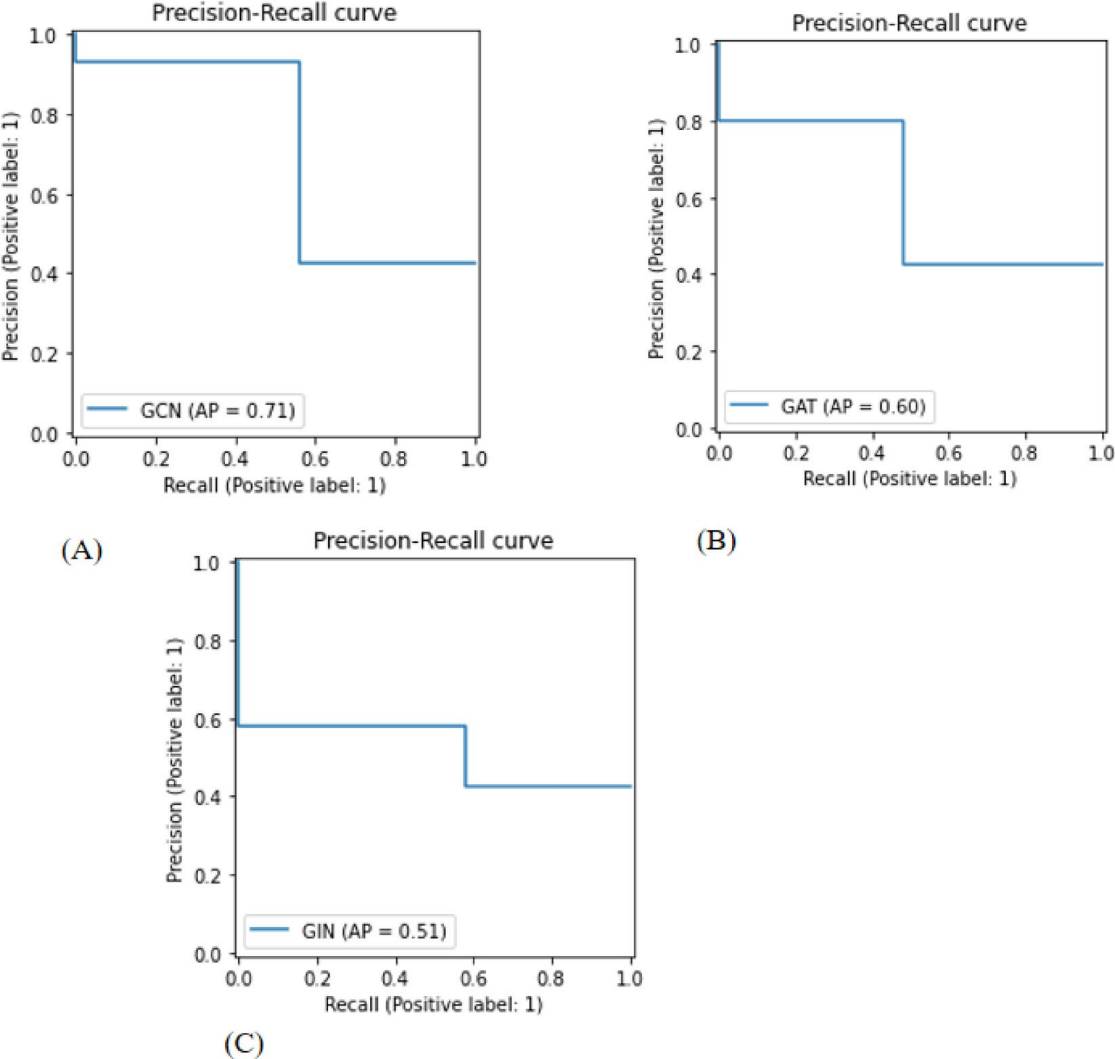
Precision-recall curve (**A**) GCN (**B**) GAT (**C**) GIN.

**Fig. 7:**
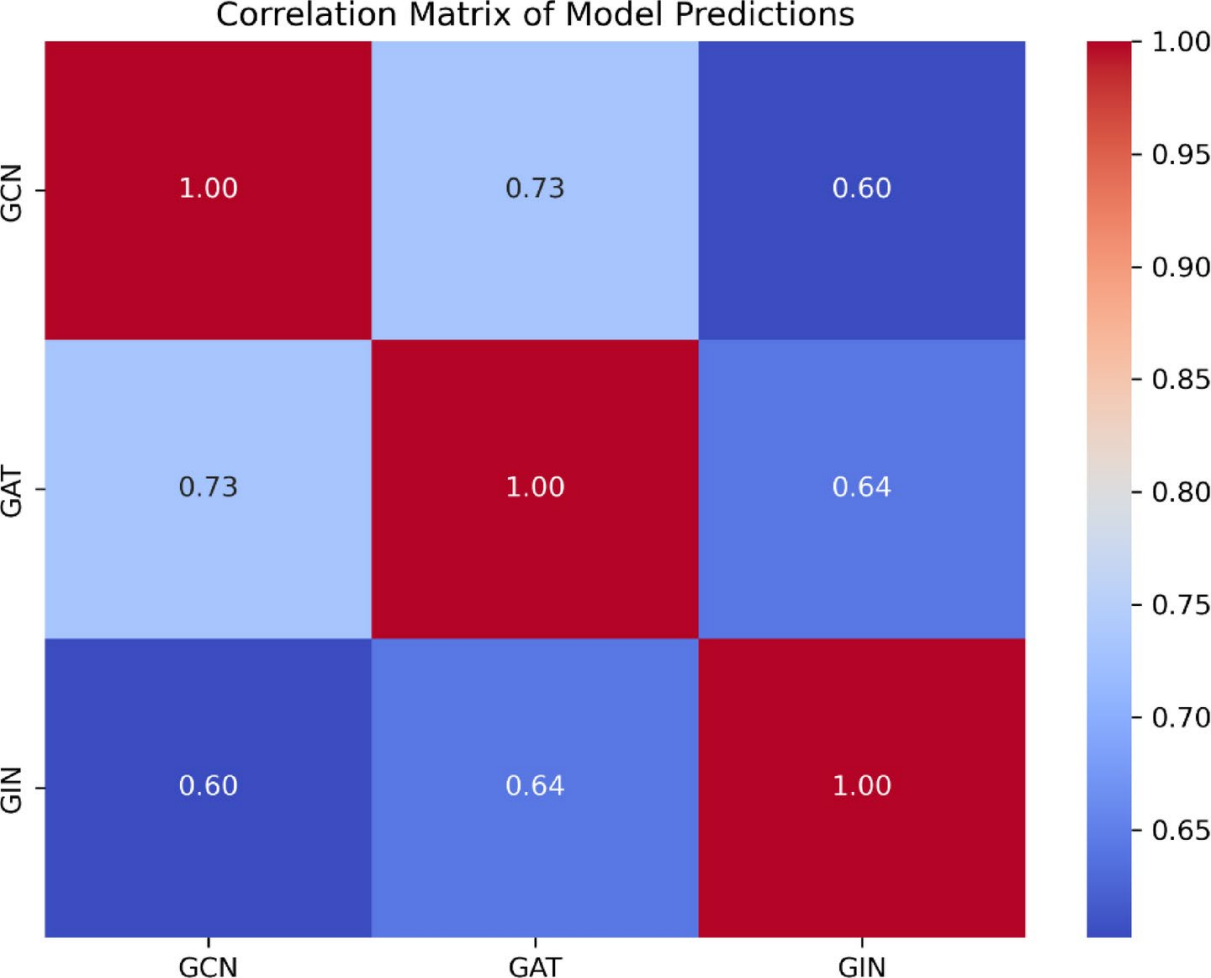
Pairwise comparison of the model’s predictions.

**Fig. 8:**
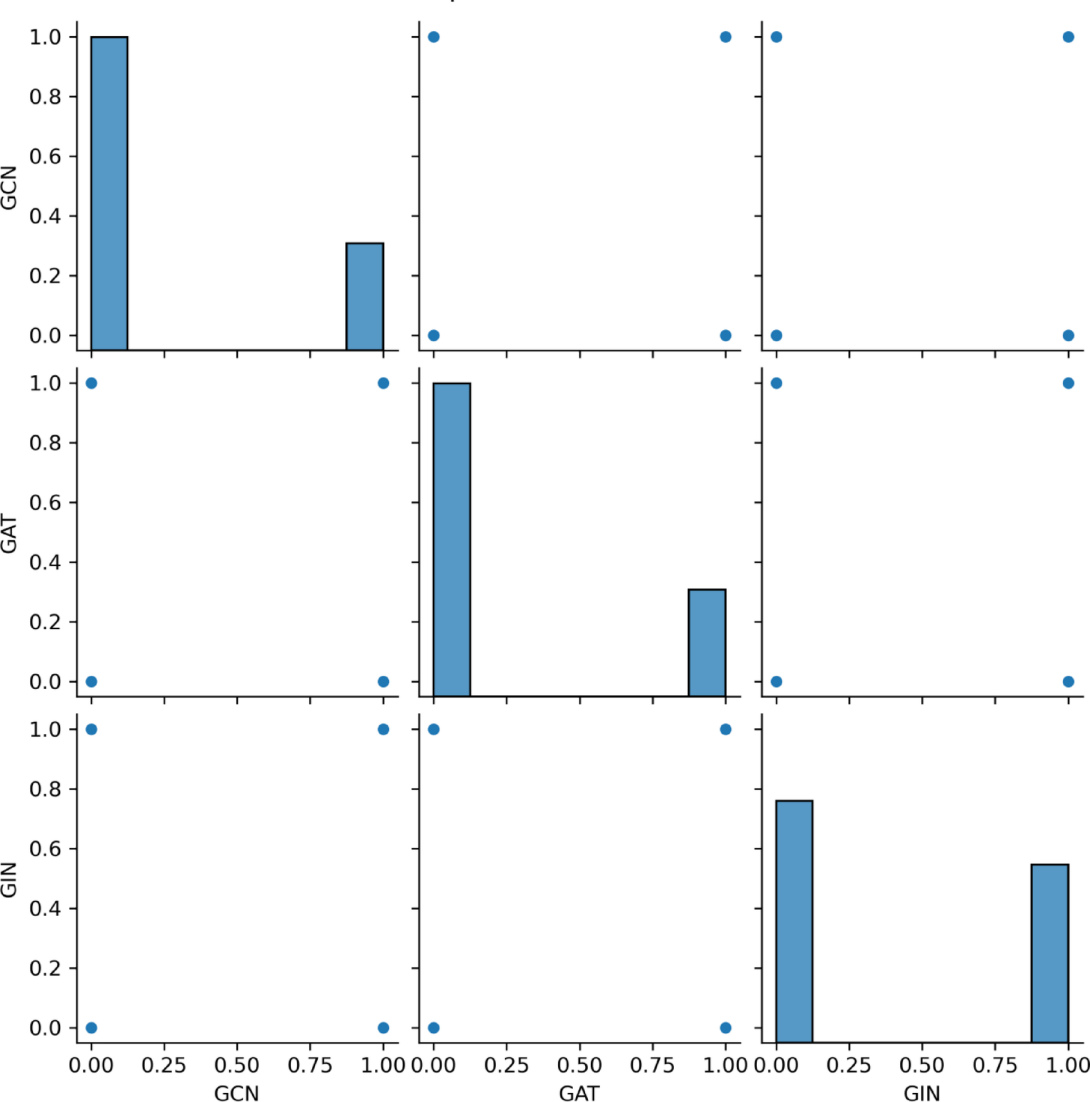
Correlation matrix of the model’s predictions.

The confusion matrices of the GCN, GAT, and GIN models in the test cohort intuitively reflected prediction accuracies (Fig. 5). Figure 6 depicts the usage of precision-recall curves to assess model performance. Figures 7 and 8 provide a pairwise comparison and correlation analysis of the models. The pairwise comparison plot visualizes the links between model predictions. The sparsity seen in scatter plots supports discrete prediction outputs, possibly due to threshold-based decision limits. The correlation matrix assesses model agreement, with correlation coefficients of 0.73 (GCN and GAT), 0.64 (GAT and GIN), and 0.60 (GCN and GIN). These moderate correlations suggest common patterns while maintaining some model independence.

## Discussion

When breast cancer patients are first diagnosed, their ALN status is distinct, and their treatment strategies vary accordingly. Patients with ALNM have worse results than those without ALNM, according to some studies^30,31^. According to the National Comprehensive Cancer Network (NCCN) guidelines, patients with ALNM should be regarded as high-risk patients and should get adjuvant chemotherapy. This is crucial information for clinicians to consider when making decisions^32^. Notably, misinterpreting the ALN status contributes to the overtreatment of patients and wastes medical resources^31^. Patients who do not have any additional risk factors and without ALNM can receive treatment solely with endocrine therapy, which results in reduced expenses and a more pleasant course of treatment^31^. Thus, it is critical to precisely differentiate the ALN status in patients with early-stage breast cancer.

In the current work, we constructed three GNN models to differentiate ALNM from non-ALNM in breast cancer patients using axillary US and clinicopathologic data. There were three notable discoveries. First, the three GNN models were able to identify ALNM from non-ALNM. Second, when comparing the three GNN models, GCN had the best prediction performance. The AUC values for GCN, GAT, and GIN were 0.77 (95% confidence interval [CI]: 0.69–0.84), 0.70 (0.62–0.77), and 0.64 (0.54–0.72), respectively. The GCN’s higher performance may be attributed to its efficient feature aggregation and smoothness limitations in message passing. Unlike GAT, which uses attention techniques to differentiate between neighboring nodes, GCN aggregates node features uniformly. While attention can help capture heterogeneous associations, it adds computational complexity and may cause weight assignment instability, especially when training with minimal data. This can restrict generalization capabilities, providing GCN an advantage in circumstances requiring global feature consistency^22–26^.

Furthermore, GCN outperforms GIN in terms of generalization across various graph architectures. GIN is intended to be highly expressive, similar to the Weisfeiler-Lehman graph isomorphism test, although its expressiveness can occasionally cause oversensitivity to slight structural alterations in the data. This can lead to less stable representations, especially in biomedical datasets such as medical US, where noise and fluctuation are prevalent. GCN, on the other hand, strikes an appropriate balance between expressiveness and robustness by enforcing a spectral-based feature smoothing effect that boosts stability and predictive performance^22–29^.

Despite having some feature representations in common with GAT and GIN, GCN also captures unique structural information that enhances its classification capacity, according to the moderate correlation (Figs. 7 and 8) between GCN and the other models. These results demonstrate how GCN’s global feature aggregation approach is advantageous in biomedical applications where robustness and consistency are critical. Third, the 10 most important factors affecting GCN’s diagnostic performance were size, location, and axillary US findings including the ratio of long axis diameter to short axis diameter < 2; diffuse cortical thickening > 3 mm; focal cortical bulge > 3 mm; eccentric cortical thickening > 3 mm; complete or partial effacement of the fatty hilum; rounded hypoechoic node; complete or partial replacement of the node with an ill-defined or irregular mass; nonhilar cortical blood flow on colour Doppler images. Most prior research on ALNM in breast cancer patients has primarily focused on single independent risk variables for ALNM, such as tumour size and grade^33,34^. The current research provides a model that is noninvasive and convenient thereby making it more advantageous than these studies.

As part of their work, Zheng et al.^19^ created and validated a model for predicting ALN status in early-stage breast cancer patients using clinicopathologic data. Their experimental results demonstrated an AUC of 0.73 and an accuracy of 0.71, indicating therapeutic relevance. In contrast to the current study, our best-performing GNN, the GCN model, had a higher AUC of 0.77 and an accuracy of 0.80. The somewhat higher AUCs and ACCs recorded in the current study could be ascribed to the model being trained on graph data.

To the best of our knowledge, this is the first study to develop a GNN model for predicting ALN status in patients with early-stage breast cancer using axillary US and clinicopathologic data, and the results are promising. As part of their investigation, Liu et al.^35^ developed a clinical model based on clinical factors for predicting ALNM in breast cancer patients. Their experimental results showed that the AUCs were 0.77, 0.78, and 0.70 for three test cohorts, while the ACCs were 0.72, 0.75, and 0.68. In contrast to the current study, our best-performing GNN, the GCN model, had an AUC of 0.77, consistent with the above study, and an accuracy of 0.80.

Furthermore, the study employed the confusion matrix (Fig. 5) to assess the model’s performance. Of the 118 patients in the test cohort, the GCN model correctly identified 28 cases (true positive) out of 50 with ALNM and 66 cases (true negative) out of 68 without ALNM (Fig. 5A). The GAT model correctly identified 24 cases (true positive) out of 50 with ALNM and 62 cases (true negative) out of 68 without ALNM (Fig. 5B). The GIN model correctly identified 29 cases (true positive) out of 50 with ALNM and 67 cases (true negative) out of 68 without ALNM (Fig. 5C).

Precision in medical diagnosis is important since it reflects the accuracy with which positive instances are identified. In particular, when predicting the presence of ALNM in breast cancer patients, a high precision indicates that the model’s identification of someone as having ALNM is highly likely to be true. This emphasis on precision is critical to avoiding unneeded therapies or interventions for people who don’t have the disease^36,37^. In the stairstep segment represented in Fig. 6, recall and precision are inversely related.

At the edges of these steps, even a slight tweak in the threshold can notably impact precision while marginally enhancing recall. By analysing the precision-recall relationship of the best-performing GNN which is the GCN model (Fig. 6A), an average precision of 0.70 was observed, indicating satisfactory performance.

While these criteria are important, they must be balanced to be effective. The F1 score, a composite statistic that combines precision and recall, is a useful tool for assessing a model’s overall performance. In this investigation, the GCN model’s F1 score was 0.70, indicating satisfactory performance.

Although the study’s GCN-based technique yielded promising findings, there are a few concerns that can be addressed in future research. Additional validation may be necessary before direct clinical adoption may occur. The data used in this study came from a single center. More evidence from multicenter is required to validate this concept before clinical implementation in the future. A larger dataset may improve the model’s robustness. Patients with multifocal breast lesions and bilateral disease were omitted since it was difficult to predict which lesion would result in ALNM and should be included in the model.

As a result, the current model can predict ALNM only for patients with a single type of breast cancer. Additional study is required to create another model for predicting ALNM in patients with multifocal breast lesions.

Finally, our findings demonstrated that GNN models performed satisfactorily in identifying ALNM in patients with early-stage breast cancer. GCN outperformed the other two GNN models. To our knowledge, this is the first study to use various GNN algorithms unique to ALNM based on axillary US and clinicopathologic data. A better-performing GNN model would aid in the identification of metastatic lymph nodes and provide a simple method for clinical and surgical decision-making in the future as suggested by the study.

## Electronic supplementary material

Below is the link to the electronic supplementary material.

Supplementary Material 1

## Data Availability

The data used in this study are available in Zheng et al. [19] published in Nature Communications and can be accessed at https://www.nature.com/articles/s41467-020-15027-z. Usage of the data is subject to the terms specified in the original publication.

## Abbreviations

ACC: Accuracy
AI: Artificial intelligence
ALND: Axillary lymph node dissection
ALNM: Axillary lymph node metastasis
AUC: Area under the curve
CNN: Convolutional neural network
ER: Estrogen receptor
GAT: Graph attention network
GCN: Graph convolutional network
GIN: Graph isomorphism network
GNN: Graph neural network
HER-2: Human epidermal growth factor receptor-2
MLP: Multi-layer perceptron
NCCN: National comprehensive cancer network
NPV: Negative predictive value
PPV: Positive predictive value
PR: Progesterone receptor
ROC: Receiver operating characteristic
SEN: Sensitivity
SLNB: Sentinel lymph node biopsy
SPEC: Specificity
US: Ultrasound
