# Supplementary material for: A comparative analysis of three graph neural network models for predicting axillary lymph node metastasis in early-stage breast cancer
Source: Sci Rep. 2025 Apr 22;15:13918. doi: 10.1038/s41598-025-97257-z (PMC12015543; doi:10.1038/s41598-025-97257-z)
Supplement: Supplementary file 1 — Supplementary Material 1 [file 41598_2025_97257_MOESM1_ESM.doc]

**Supplementary Method: Evaluation metrics employed in this study**

The models' performance on both the training and validation datasets was evaluated using metrics such as the area under the curve (AUC), confusion matrix, precision, sensitivity, specificity, negative predictive value (NPV), positive predictive value (PPV), accuracy, F1 score, and other standard clinical statistics. In ALNM prediction, The ROC curve plots the true positive rate (sensitivity) against the false positive rate (1 – sensitivity), and the AUC can be calculated. The accuracy, in the context of ALNM prediction, gauges the model's overall accuracy in predicting the presence or absence of ALNM in individuals with breast cancer. The accuracy can be calculated from Eqn (4)[1]. Precision in the context of ALNM prediction refers to the ratio of accurately predicted ALNM cases to all cases that the model presented as having ALNM. Eqn. (2) determines the precision [1]. The F1 score (Eqn. 3) balances precision and recall in the context of ALNM prediction by offering a harmonic mean that encompasses both false positives and false negatives [2]. Recall or sensitivity (Eqn. 1 and 5) for ALNM prediction represents the ratio of correctly predicted cases of ALNM out of all actual cases of ALNM based on the true positive rate, showing a model's capacity to correctly identify all people who have ALNM [2].

In the context of ALNM prediction, specificity (Eqn. 6) is described in a variety of ways, including a model's capacity to detect true negatives, being based on the true negative rate, and properly identifying those who do not have ALNM. These evaluation metrics play a crucial role in assessing the efficiency and effectiveness of machine learning models. In Equations 1, 2, 4, 5, 6, 7, and 8, *TP* represents the number of true positive predictions (correctly predicted positive ALNM), *FP* represents the number of false positive predictions (incorrectly predicted positive ALNM), *TN* represents the number of true negative predictions (correctly predicted negative ALNM), and *FN* represents the number of false negative predictions (incorrectly predicted negative ALNM). These metrics provide valuable insights into the accuracy and performance of ALNM predictions.

(1)

(2)

(3)

(4)

(5)

(6)

(7)

(8)

**Supplementary References**

[1] Md. A. Islam, Md. Z. H. Majumder, and Md. A. Hussein, “Chronic kidney disease prediction based on machine learning algorithms,” *J. Pathol. Inform.*, vol. 14, p. 100189, Jan. 2023, doi: 10.1016/j.jpi.2023.100189.

[2] Md. Ziaul Hasan Majumder, Md. Abu Khaer, Md. J. Nayeen Mahi, Md. Shaiful Islam Babu, and S. K. Aditya, “Decision Support Technique for Prediction of Acute Lymphoblastic Leukemia Subtypes Based on Artificial Neural Network and Adaptive Neuro-Fuzzy Inference System,” in *Inventive Systems and Control*, vol. 204, V. Suma, J. I.-Z. Chen, Z. Baig, and H. Wang, Eds., in Lecture Notes in Networks and Systems, vol. 204. , Singapore: Springer Singapore, 2021, pp. 539–554. doi: 10.1007/978-981-16-1395-1_40.
